# Embryos of the Viviparous Dermapteran, *Arixenia esau* Develop Sequentially in Two Compartments: Terminal Ovarian Follicles and the Uterus

**DOI:** 10.1371/journal.pone.0064087

**Published:** 2013-05-08

**Authors:** Waclaw Tworzydlo, Elzbieta Kisiel, Szczepan M. Bilinski

**Affiliations:** Department of Developmental Biology and Morphology of Invertebrates, Institute of Zoology, Jagiellonian University, Krakow, Poland; University of Otago, New Zealand

## Abstract

Three main reproductive strategies have been described among insects: most common oviparity, ovoviviparity and viviparity. In the latter strategy, the embryonic development takes place within the body of the mother which provides gas exchange and nutrients for embryos. Here we present the results of histological and EM analyses of the female reproductive system of the viviparous earwig, *Arixenia esau*, focusing on all the modifications related to the viviparity. We show that in the studied species the embryonic development consists of two “physiological phases” that take place in two clearly disparate compartments, i.e. the terminal ovarian follicle and the uterus. In both compartments the embryos are associated with synthetically active epithelial cells. We suggest that these cells are involved in the nourishment of the embryo. Our results indicate that viviparity in arixeniids is more complex than previously considered. We propose the new term “pseudoplacento-uterotrophic viviparity” for this unique two-phase reproductive strategy.

## Introduction

Three main reproductive strategies have been described among insects: oviparity, viviparity and ovoviviparity [1]. The vast majority of studied insect species is characterized by oviparity. Females of oviparous species lay down eggs in a safe habitat where embryogenesis takes place. During development, the embryos use the reserve materials (yolk proteins and lipids) stored during oogenesis in the oocyte cytoplasm, and are surrounded by protective coverings, the vitelline envelope and the chorion (see [1]–[3] for a review). The second strategy, i.e. viviparity, is relatively rare among insects. In this reproductive mode, the embryonic development takes place within the mother's body which provides gas exchange and, what is more important, nourishments for the embryos [4]–[6]. In some insect groups, less advanced or “transitional” reproductive mode, the ovoviviparity has been reported. In this case, the embryos develop inside eggs that are retained in the mother's body until they are ready to hatch [1]. Hatching occurs just after oviposition or the larvae hatch inside the mother's reproductive system and are later deposited or “born”. The latter process is termed, the larviposition. Although, the ovoviviparity is deceptively similar to viviparity it does not require any nourishment of the embryos/larvae by the mother, as the unborn offspring use the reserve materials accumulated in the egg cytoplasm.

Earwigs (Dermaptera) is a relatively small insect order with about 2200 living species traditionally classified in three suborders: two epizoic groups, the Hemimerina and Arixeniina, and free-living Forficulina [7], [8]. Although the classification of extant dermapterans is still under debate, recent morphological and histological studies imply that both epizoic groups should be included in the most derived dermapteran subgroup, the Eudermaptera (Spongiphoridae + Chelisochidae + Forficulidae), and do not constitute separate suborders (see [9], [10] for further discussion of dermapteran classification and cladograms therein). According to this concept, clearly disparate morphology of hemimerids and arixenids is interpreted as a result of ectoparasitic (epizoic) life.

The majority of dermapteran species is, like most insects, oviparous. The females lay eggs in characteristic nests and, what is worth noting, in some cases they look after the offspring showing a simple example of a maternal care. In contrast, two epizoic groups (the Arixeniina and Hemimerina) are viviparous [4], [11]. It was suggested that in these groups, viviparity evolved to provide nymphs with an immediate contact with the preferred host and to accelerate the life cycle [4], [11]. It is interesting to note here that the third aforementioned reproductive strategy, the ovoviviparity, has also been reported in dermapterans [12], [13]. Significantly, the ovoviviparous species belong to the derived family Spongiphoridae (the Eudermaptera), and are free-living.

In our previous EM study, we have shown that in *Arixenia esau*, female reproductive system consists of merositic-polytrophic ovaries, strongly dilated lateral oviducts (the uteri) and a single common oviduct leading to the female genital opening [10]. Our analyses have additionally indicated that the ovaries and developing oocytes of *A. esau* exhibit unique mixture of characters shared with derived earwigs (Forficulidae) and important adaptations to the viviparity. In this publication, we describe the morphology and ultrastructure of the uteri and terminal ovarian follicles of the same species. We focus on all the modifications related to the intrauterine and “intrafollicular” development of embryos. Our studies are in line with classical histological analyses of the related species *Arixenia jacobsoni* [4], providing new and interesting data on the morphology and possible functioning of the female reproductive system of the Arixeniina.

## Materials and Methods

### Ethics statement

No specific permits were required for the described field studies. All locations of insect collecting were neither privately-owned nor protected in any way. *Arixenia esau* is not a protected or endangered species.

### Animals

The adult females of *Arixenia esau* Jordan 1909 were collected from the walls of small caves and hollow trees (inhabited by colonies of *Cheiromeles torquatus* bats) in Bintulu District area, Sarawak, Malaysia in the second half of February 2010. For this study 11 specimens were used. Multiple specimens were examined for each developmental stage. The female reproductive systems were dissected and fixed either in 4% formaldehyde or in 2.5% glutaraldehyde in 0.1 M phosphate buffer.

### Light and electron microscopy

Dissected samples were rinsed and postfixed in 1% osmium tetroxide. After dehydration in the series of ethanol and acetone the material was embedded either in Glycid Ether 100 (formerly known as Epon 812) (Serva, Heidelberg, Germany) or Histocryl (Agar Scientific Ltd., Stansed, Essex, UK). Semi-thin sections (0.7 μm thick) were stained with 1% methylene blue and examined under a Leica DMR microscope (Heidelberg, Germany) (LM). Ultrathin sections (80 nm thick) were contrasted with uranyl acetate and lead citrate according to standard protocols and analyzed with a Jeol JEM 100 SX transmission electron microscope (TEM) at 80 kV.

### Scanning electron microscopy

For the SEM, the material was fixed and postfixed as described above. After dehydration in graded series of ethanol, the material was critical-point dried, coated with gold and examined with a JSM 5410 scanning electron microscope at 25 kV. Some specimens analyzed in SEM, were immersed in 70% ethanol, dehydrated and embedded in Histocryl as described above.

### AgNOR technique

Dating from the early days of Ramon y Cajal and Golgi, it was known that impregnation with silver nitrate results in selective staining of the interphase nucleolus as well as the Golgi apparatus [14]–[16]. More recent papers showed that silver salts are reduced also by derivatives of the Golgi apparatus, e.g. cortical granules and acrosomal vesicles [17], [18]. In this context, we employed this procedure to examine whether epithelial cells of the uterus are synthetically active or not. Silver impregnation was performed on semi-thin Histocryl sections, according to the method described by Howell and Black [19] modified by Bilinski and Bilinska [20]. The sections were stained with a 1∶2 mixture of 2% gelatin in 1% formic acid and 50% silver nitrate (Sigma, St. Louis, MO, USA). The staining was carried out for 15 minutes in 37°C. After rinsing with distilled water the slides were analyzed with a Leica DMR microscope.

### DNA detection

Ovarioles were fixed, dehydrated in the series of ethanol and embedded in Histocryl as described above. Semi-thin sections (0.7 µm thick) were stained with DAPI (1 µg/ml; Sigma, St. Louis, MO, USA) in the darkness, for 40 minutes, and analyzed with a Leica DMR fluorescence microscope (FM), equipped with appropriate filters [21].

## Results

### Gross morphology of the female reproductive system and the ovariole

**Figure 1:**
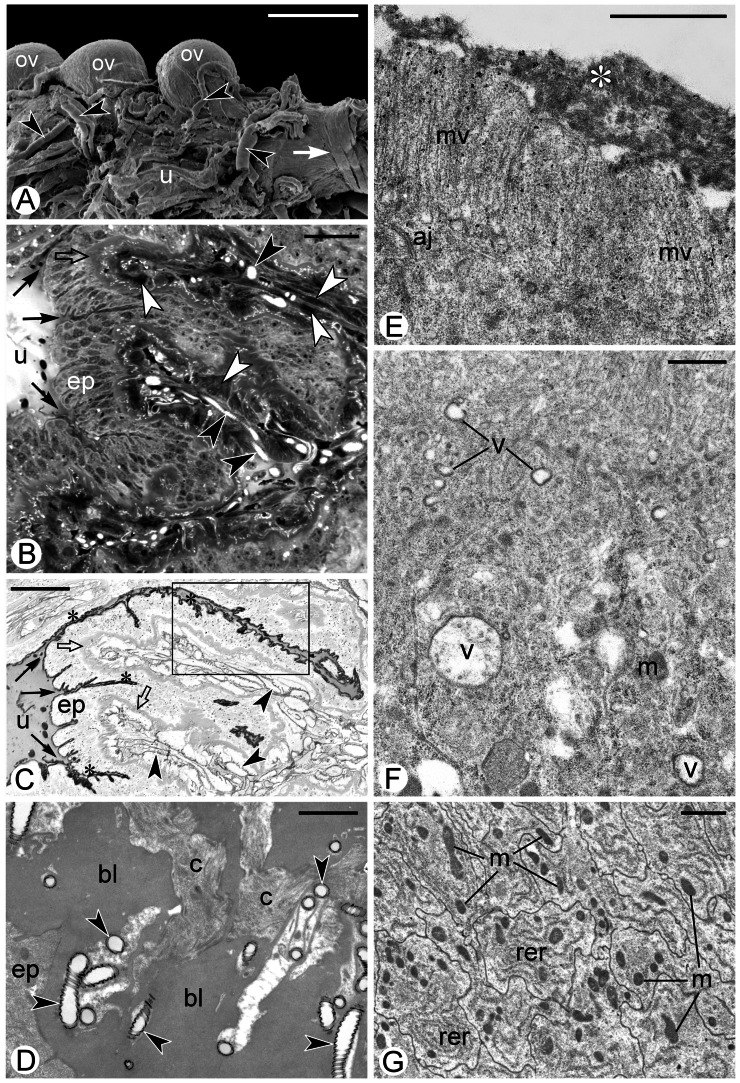
Morphology of the female reproductive system. (**A**) Three ovarioles (ov) attached to the uterus (u). White arrow indicates circumferential muscle fibers surrounding the posteriormost section of the uterus. Tracheal branches (black arrowheads). SEM, scale bar: 0.5 mm. (**B, C**) Transverse section through the uterus (fragment). Note folded epithelium (ep) lining the uterus (u). Basement lamina (empty arrows), muscle fibers (white arrowheads), tracheal branches (black arrowheads), grooves separating ridges of the epithelium (arrows), black asterisks in (C) indicate argyrophylic material on the cell surface. Boxed fragment in (C) is enlarged in Figure 2. (B) Semi-thin section stained with methylene blue. (C) Semi-thin section stained with AgNOR technique. LM, scale bar: 24 µm. (**D**) Basement lamina (bl) supporting epithelial cells (ep) of the uterus. The lamina is penetrated by canals (c) containing tracheal branches (black arrowheads) immersed in filamentous material. TEM, scale bar: 1 µm. (**E, F**) Apical compartment of an epithelial cell lining the uterus. Note large secretory vacuoles (v), fibro-granular material covering the tips of the microvilli (white asterisk) and an adherens junction (aj) connecting membranes of neighboring cells. Mitochondria (m). TEM, scale bar: 1 µm. (**G**) Basal compartments of epithelial cells of the uterus. Mitochondria (m), elements of rough endoplasmic reticulum (rer). Note that plasma membranes are folded and closely adjoined. TEM, scale bar: 1 µm.

The reproductive system of the *Arixenia esau* females consists of paired ovaries, paired lateral oviducts and a single common oviduct that leads to a female genital opening [9], [10]. Each ovary is composed of three elongated, synchronously developing ovarioles that are attached to the one of the lateral oviducts (Figure 1A). The lateral oviducts are markedly dilated (Figure 1A) and contain developing embryos, and therefore will be termed the uteri. The ovarioles as well as the uteri are surrounded by a thick basement lamina and well developed sheath containing several layers of muscle fibers and tracheal branches (Figure 1A).

In adult females, the ovarioles consist of 3 elements: an anterior terminal filament, germarium and a posterior vitellarium [10]. The vitellarium comprises 2–3 previtellogenic ovarian follicles and a single, distinctly larger terminal follicle. The latter contains either vitellogenic/postvitellogenic oocytes [10] or embryos in various developmental stages.

### The uterus

The wall of the uterus is lined with one-cell-thick epithelium resting on a thick (up to 7 µm) basement lamina (Figure 1B, C, D). The epithelial layer and associated lamina are extensively folded that leads to the formation of a number of wide ridges (interspersed by deep grooves) on both, internal and external (i.e. adluminal and haemocelic) surfaces of the uterus (Figure 1B, C, arrows). Measurements performed on isolated organs and semi-thin sections, have shown that the full perimeter of the “stretched” epithelium is 3–4 times larger than the perimeter of the uterus. The epithelial lining of the uterus is supported by a prominent layer of muscle fibers and tracheal branches (Figure 1B, white and black arrowheads, respectively). Both muscle fibers and tracheal branches extend radially and penetrate into the heamocelic grooves of the epithelial layer (Figure 1B, C), forming an elastic “scaffold” of the organ. The posteriormost part of the uterus narrows (Figure 1A) and leads to the common oviduct. This part is additionally surrounded by 3–4 circumferential muscle fibers (Figure 1A, white arrow).

### Epithelial cells of the uterus

**Figure 2:**
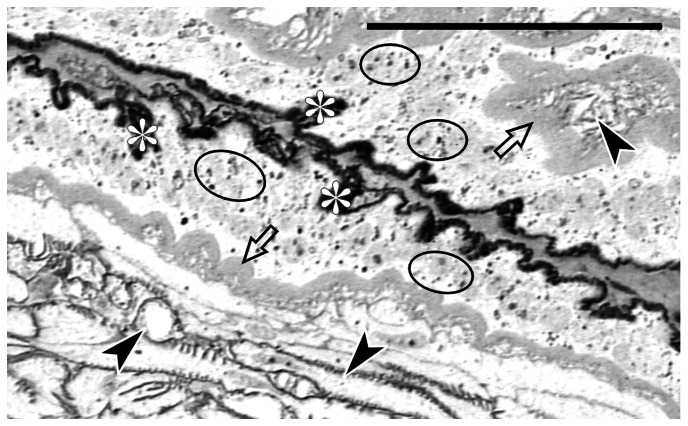
Secretory activity of the uterus epithelial cells. Transverse section through the infolding of the uterus wall (boxed fragment of Figure 1C). Two closely opposed epithelial layers are visible. Note argyrophylic secretory vacuoles in the cytoplasm of epithelial cells (encircled). Basement lamina (empty arrows), tracheal branches (black arrowheads), argyrophylic material on the cell surface (white asterisks). Semi-thin section stained with AgNOR technique. LM, scale bar: 24 µm.

The epithelial cells lining the uterus are slender and markedly elongated (Figure 1B). Their apical surfaces are equipped with long and parallel microvilli (Figure 1E). The tips of the microvilli are covered with a layer of electron dense fibro-granular material (Figure 1E, asterisk). Staining with silver nitrate has revealed that this material contains argyrophilic proteins (Figures 1C and 2, asterisks). The apical cytoplasm of the epithelial cells contains free ribosomes, individual mitochondria and numerous spherical, secretory vesicles (Figure 1E, F). The membrane of the vesicles is lined with a thin layer of electron-dense material (Figure 1F). Careful examination of sections stained with silver nitrate has shown that the secretory vesicles (as the material covering the microvilli) are argyrophilic (Figure 1C and Figure 2, encircled). This observation suggests that the dense material covering the microvilli tips is synthesized within, and secreted from uterine epithelial cells.

Between apical plasma membranes of neighboring cells, short adherens junctions have been occasionally found (Figure 1E). The nuclei reside in the cell centers, and separate the apical cytoplasm from the rest of the cell. Analysis of cross sections has revealed that plasma membranes surrounding basal cytoplasm compartments are folded and closely adjoined (Figure 1G). In the basal cytoplasm elongated cisternae of rough endoplasmic reticulum (RER) and small spherical or slightly elongated mitochondria (Figure 1G) reside. The basal cell surfaces rest on a thick and homogenous basal lamina (Figure 1D). The lamina is perforated by branching canals that contain the thinnest tracheal branches immersed in a filamentous material of medium electron density (Figure 1D).

### Terminal follicles and initial stages of development

**Figure 3:**
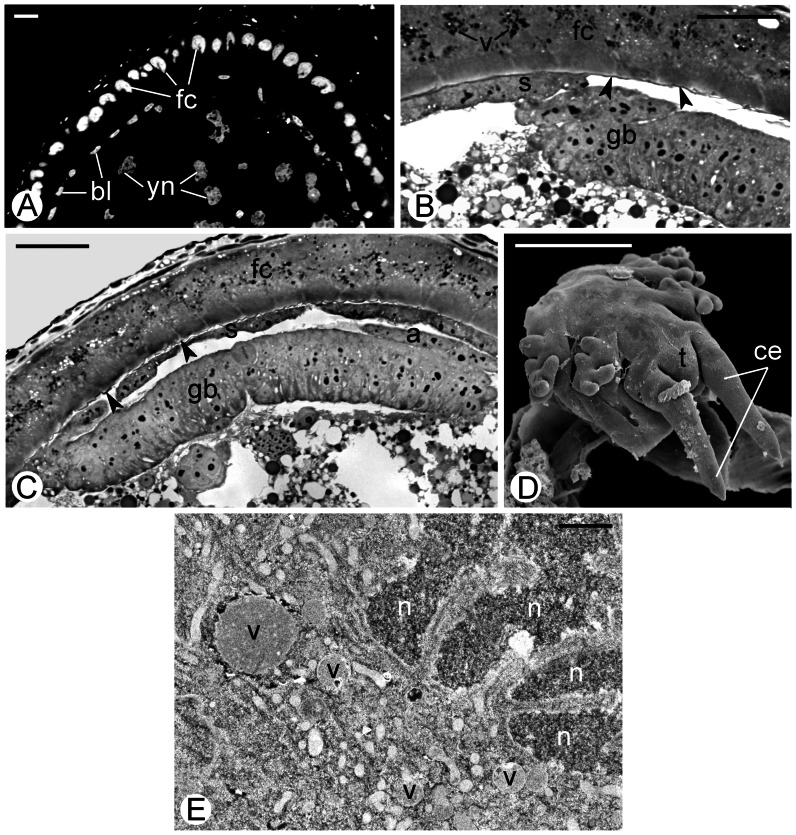
Embryonic development. (**A**) Fragment of a young embryo (cross section; blastoderm stage) developing inside a terminal ovarian follicle. DAPI staining. Note that the nuclei of follicular cells (fc) are substantially larger and “brighter” than blastoderm nuclei (bl). Yolk nuclei (yn). FM, scale bar: 24 µm. (**B, C**) Fragment of an older embryo (cross section; germ band stage) inside a terminal follicle. Note the germ band (gb) surrounded by the follicular epithelium (fc) and two embryonic envelopes, serosa (s) and amnion (a). The follicular epithelium is separated from the embryo by a thin structureless eggshell (arrowheads). LM, scale bar: 24 µm. (**D**) Posterior part of the embryo dissected from the lumen of the uterus. Note well developed cerci (ce) and the last abdominal segment (telson, t). SEM, scale bar: 0.5 mm. (**E**) Follicular cell (fragment) surrounding the blastoderm stage embryo. Note highly folded envelope surrounding irregularly shaped nucleus (n) and large secretory vacuoles (v). TEM, scale bar: 1 µm.

As it has been shown in classical embryological analyses, substantial part of the embryonic development (at least to the final phase of the dorsal closure) of *Arixenia* takes place inside terminal ovarian follicles and within one-cell-thick follicular epithelium that in younger specimens surrounds developing oocytes [4]. We show that the cells of this epithelium are large (Figure 3C) and comprise irregularly shaped nuclei with deeply folded nuclear envelope (Figure 3A, E). The cytoplasm of follicular cells is densely packed with free ribosomes, rod-like mitochondria and secretory vesicles (Figure 3B, E). The vesicles are roughly spherical and filled with fine granular material of medium electron density (Figure 3E).

The embryos do not adhere directly to the epithelium, and are separated from it by a thin eggshell, inadequately termed by Hagan [4] the periplasmic membrane (Figure 3B, C, arrowheads). For the detailed description of the eggshell see [10].

Inside ovarian follicles of the analyzed specimens, we have found relatively young embryos in blastoderm stage (Figure 3A) as well as more advanced ones with already differentiated germ bands surrounded by embryonic envelopes, the serosa and the amnion (Figure 3B, C). Analysis of sections stained with DAPI has shown that the blastoderm nuclei are distinctly smaller and produce fainter fluorescence than these of the follicular cells (Figure 3A). This observation implies that the nuclei of the follicular cells contain much more DNA and are presumably polyploid.

After a definitive dorsal closure, embryos “move down to the another portion of the reproductive tract” [4], i.e. the uterus (Figure 3D), where they develop until the birth of the offspring (compare the Discussion).

## Discussion

Viviparous reproduction in insects is different from the well-known mammalian system in that it lacks a typical placenta, an organ transferring nutrients from the mother to the developing embryo. Hagan [4] and Retnakaran and Percy [6] classified insect viviparity into 4 main types.

1. Ovoviviparity, at present regarded as a separate type of a reproductive strategy (see the Introduction);
2. Adenotrophic viviparity. Fertilized eggs develop inside swollen part of the common oviduct, termed the uterus. After hatching, the larvae are retained in the uterus where they feed (orally) on a secretion of a highly modified accessory gland, termed the milk gland. This type of the viviparity has been described in the dipteran families Glossinidae and Hippoboscidae (see [4], [22]–[25] for details).
3. Haemocoelous viviparity. Embryos and larvae develop in the haemocoel, and take up nutrients directly from the haemolymph. Haemocoelous viviparity has been found in strepsipterans and dipterans from the family Cecidomyiidae [4], [6] that possess highly modified “dispersed” ovaries (see [26]–[31] for a review).
4. Pseudoplacental viviparity. Embryos and larvae develop inside terminal ovarian follicles and are nourished by highly active follicular epithelium that in earlier stages surrounded developing oocytes. This epithelium (or its specialized areas) is thickened and termed by Hagan “the pseudoplacenta” [4]. Pseudoplacental viviparity has been described in aphids [32], certain psocopterans [6], cockroaches [33]–[35] and both epizoic dermapteran groups, the Arixeniina and Hemimerina [4], [6]. It is interesting to note that the “placenta-like structure” functions also during development of the endoparasitic wasp, *Aphidius ervi* [36]. Here, the placenta invades the tissues of the host (viviparous aphid) and transfers nutrients prepared for the host offspring to the endoparasitic embryo [36].

Our studies clearly show that the pseudoplacental viviparity, at least in Arixeniina, is more complex than considered earlier [6]. Initial stages of the development indeed take place inside the terminal ovarian follicles as described by Hagan [4], however later, the embryos are transferred to the uteri where they remain until the birth of the offspring. Thus, the embryonic development of *A. esau* consists of two “physiological phases” that take place in two morphologically disparate compartments. It should be stressed here, that the “intrauterine” phase of embryonic development has not been analyzed to date; in his fundamental work, Hagan mentioned only: “…the last three (embryos)…seem to have moved down into another portion of the reproductive tract, probably into the paired oviduct” [4, p. 288].

In contrast to the ovoviviparity and haemocoelous viviparity, the adenotrophic and the pseudoplacental strategies require essential modifications of the female reproductive tract, most notably transformation of the oviduct (or its part) into the uterus. At the EM level, these modifications have been described only in tsetse flies (Diptera: Glossinidae). In this group, the uterus is single and lined with squamous epithelial cells covered with thin cuticular intima [37]; these observations conclusively indicate that it originates from the ectoderm. The situation in *A. esau* is clearly different. Here, the uteri apparently originate from the mesoderm, as they are lined with columnar epithelial cells that are not associated with intima but equipped with microvilli. Irrespective of clearly disparate origin, the uteri of *A. esau*, and those of tsetse flies are morphologically similar. They are covered with several muscle fibers and tracheal branches that provide elasticity of the organ and gas exchange for the embryo, respectively.

We show that in *A. esau* the embryos develop in contact with two populations of epithelial cells, i.e. the follicular cells (during the first phase of the embryogenesis) and the epithelial cells lining the uterus (during the second one). The follicular cells are large, polyploid, and, what is especially indicative, contain prominent secretory vacuoles even after termination of oogenesis and deposition of the eggshell. The epithelial cells of the uterus also have the appearance of synthetically active cells; they comprise conspicuous secretory vacuoles and are covered with electron-dense granular material that, in our opinion, might be essential in the nourishment of the embryos. From these results emerges that the embryos of *A. esau* not only develop in two different compartments but are (sequentially) nourished there by two separate populations of epithelial cells. This notion is supported by the following findings:

1. The oocytes of the species studied contain only small (i.e. insufficient to support embryogenesis) amount of reserve materials [4], [10];
2. The eggshell (the periplasmic membrane in [4]) is thin and presumably permeable for nutrients secreted from the follicular cells [4], [10];
3. Throughout embryogenesis, the embryos develop inside the reproductive system of the mother; they do not have a direct contact with the haemocoel, and therefore, cannot take up nutrients directly from the haemolymph [4], [6].

In conclusion, we propose the new term “the psudoplacento-uterotrophic viviparity” for the complex, two-phase reproductive strategy found in *A. esau*.

## References

[1] Wheeler D (2003) Reproduction. Female. In: Resh WH, Carde RT, editors. Encyclopedia of Insects. Academic Press. 991–993.

[2] Kunkel JG, Nordin JH (1985) Yolk proteins. In: Kerkut GA, Gilbert LI, editors. Comprehensive Insect Physiology, Biochemistry and Pharmacology. Pergamon Press. 83–111.

[3] Margaritis LH (1985) Structure and physiology of the eggshell. In: Kerkut GA, Gilbert LI, editors. Comprehensive Insect Physiology, Biochemistry and Pharmacology. Pergamon Press. 153–226.

[4] Hagan HR (1951) Embryology of viviparous insects. Ronald Press, New York 472p.

[5] Andrews RM, Rose BR (1994) Evolution of viviparity. Constrains on egg retention. Physiol Zool 67: 1006–1024.

[6] Retnakaran A, Percy J (1985) Fertilization and special modes of reproduction. In: Kerkut GA, Gilbert LI, editors. Comprehensive Insect Physiology, Biochemistry and Pharmacology. Pergamon Press. 231–293.

[7] Popham EJ (1985) The mutual affinities of the major earwig taxa (Insecta, Dermaptera). Z Zool Syst Evol 23: 199–214.

[8] Haas F, Kukalova-Peck J (2001) Dermaptera hindwing structure and folding: new evidence for superordinal relationship within Neoptera (Insecta). Eur J Entomol 98: 445–504.

[9] Tworzydlo W, Bilinski SM, Kocarek P, Haas F (2010) Ovaries and germline cysts and their evolution in Dermaptera (Insecta). Arthropod Struct Dev 39: 360–368. 20566316 10.1016/j.asd.2010.05.004

[10] Tworzydlo W, Lechowska-Liszka A, Kocarek P, Bilinski SM (2012) Morphology of the ovarioles and the mode of oogenesis of *Arixenia esau* support the inclusion of Arixeniina to the Eudermaptera. Zool Anz (in press; doi: 10.1016/j.jcz.2012.11.002).

[11] Nakata S, Maa T (1974) A review of the parasitic earwigs. Pacific Insects 16: 307–374.

[12] Herter K (1943) Zur Fortpflanzungsbiologie eines lebendgebäranden Ohrwurms (*Prolabia arachidis* Yersin). Z Morphol Tiere 40: 158–180.

[13] Kocarek P (2009) A case of viviparity in a tropical non-parasitic earwig (Dermaptera, Spongiphoridae). Trop Zool 22: 237–241.

[14] Ramon y Cajal S (1903) Un sencillo metodo de coloracion del reticulo protoplasmico y sus efectos en los diversos organos nerviosos. Trab Lab Invest Biol 2: 129–221.

[15] Thompson SW (1966) Selected histochemical and histopathological methods. Charles C Thomas Publisher 1639p.

[16] Ochs RL (1997) Methods used to study structure and function of the nucleolus. Methods Cell Biol 53: 303–321. 10.1016/s0091-679x(08)60884-5 9348514

[17] Sousa M, Azevedo C (1987) Silver staining of the cortical reaction in oocytes of *Marthasterias glacialis* (Echinodermata, Asteroidea). Can J Zool 65: 2607–2611.

[18] Sousa M, Azevedo C (1988) Comparative silver staining analysis on spermatozoa of various invertebrate species. Int J Invert Reprod Dev 13: 1–8.

[19] Howell WM, Black DA (1980) Controlled silver-staining of nucleolus organizer regions with protective colloidal developer: a 1-step method. Experientia 36: 1014. 6160049 10.1007/BF01953855

[20] Bilinski SM, Bilinska B (1996) A new version of the Ag-NOR technique. A combination with DAPI staining. Histochem J 28: 651–656. 8910036 10.1007/BF02331386

[21] Tworzydlo W, Jablonska A, Kisiel E, Bilinski SM (2005) Differing strategies of patterning of follicular cells in higher and lower brachycerans (Diptera: Brachycera). Genesis 43: 49–58. 16100706 10.1002/gene.20155

[22] Tobe SS, Langley PA (1978) Reproductive physiology of *Glossina* . Annu Rev Entomol Res 23: 283–307. 10.1146/annurev.en.23.010178.001435 343707

[23] Tobe SS, Davey KG, Huebner E (1973) Nutrient transfer during the reproductive cycle in *Glossina austeni* Newst.: histology and histochemistry of the milk gland, fat body and oenocytes. Tissue Cell 4: 633–650. 10.1016/s0040-8166(73)80050-3 4129183

[24] Attardo GM, Guz N, Stickler-Dinglasan P, Aksoy S (2006) Molecular aspects of viviparous reproductive biology of the tsetse fly (*Glossina morsitans morsitans*): regulation of yolk and milk gland protein synthesis. J Insect Physiol 52: 1128–1136. 17046784 10.1016/j.jinsphys.2006.07.007 PMC1779500

[25] Attardo GM, Lohs C, Heddi A, Alam UH, Yildirim S, et al (2008) Analysis of milk gland structure and function in *Glossina morsitans*: milk protein production, symbiont populations and fecundity. J Insect Physiol 54: 1136–1242. 10.1016/j.jinsphys.2008.06.008 PMC2613686 18647605

[26] Buning J (1998) Reductions and new inventions dominate the oogenesis of Strepsiptera (Insecta). Int J Morphol Embryol 27: 3–8.

[27] Kathirithamby J, Carcupino M, Mazzini M (1990) Ovarian structure in the order Strepsiptera. Frust Entomol 13: 1–8.

[28] Buning J (1994) The insect ovary. Ultrastructure, Previtellogenic Growth and Evolution. Chapman & Hall 400p.

[29] Meier R, Kotrba M, Ferrar P (1999) Ovoviviparity and viviparity in the Diptera. Biol Rev 74: 199–258.

[30] Schupbach PM, Camenzind R (1983) Germ cell lineage and follicle formation in paedogenetic development of *Mycophila speyeri* (Diptera: Cecidomyiidae). Int J Insect Morphol Embryol 12: 211–224.

[31] Junquera P (1984) Oogenesis in paedogenetic dipteran insect under normal conditions and after experimental elimination of the follicular epithelium. Roux's Arch Dev Biol 193: 197–204. 10.1007/BF01260339 28305214

[32] Bermingham J, Wilkinson TL (2009) Embryo nutrition in parthenogenetic viviparous aphids. Physiol Entomol 34: 103–109.

[33] Holbrook GL, Schal C (2004) Maternal investment affects offspring phenotypic plasticity in a viviparous cockroach. PNAS 101: 5595–5597. 15064397 10.1073/pnas.0400209101 PMC397435

[34] Williford A, Stay B, Bhattacharya D (2004) Evolution and novel function: nutritive milk in the viviparous cockroach *Diploptera punctata* . Evol Dev 6: 67–77. 15009119 10.1111/j.1525-142x.2004.04012.x

[35] Younsteadt E, Fan Y, Stay B, Schal C (2005) Cuticular hydrocarbon synthesis and its maternal provisioning to embryos in the viviparous cockroach *Diploptera punctata* . J Insect Physiol 51: 803–809. 15899497 10.1016/j.jinsphys.2005.03.008

[36] Sabri A, Hance T, Leroy PD, Frere I, Haubruge E, et al.. (2011) Placenta-like structure of the aphid endoparasitic wasp *Aphidius ervi*: a strategy of optimal resources acquisition. PLOS One 6, e18847. 10.1371/journal.pone.0018847 PMC3079733 21526196

[37] Pellegrini A, Bigliardi E, Bechi N, Paulesu L, Lehane MJ, et al (2011) Fine structure of the female reproductive system in a viviparous insects, *Glossina morsitans morsitans* (Diptera, Glossinidae). Tissue Cell 43: 1–7. 21094964 10.1016/j.tice.2010.10.005

